# A plea to provide best evidence in trials under sample-size restrictions: the example of pioglitazone to resolve leukoplakia and erythroplakia in Fanconi anemia patients

**DOI:** 10.1186/s13023-017-0655-8

**Published:** 2017-05-25

**Authors:** Florian Lasch, Kristina Weber, Mwe Mwe Chao, Armin Koch

**Affiliations:** 1 Department of Biostatistics, Hannover Medical School, Carl-Neuberg Strasse 1, 30165 Hannover, Germany; 2 Department of Pediatric Hematology Oncology, Hannover Medical School, Hannover, Germany

**Keywords:** Randomization, Single-arm trial, RCT, Rare disease, Historical control, Fanconi anemia, Study planning, Evidentiary standards

## Abstract

In planning a clinical trial for demonstrating the efficacy of pioglitazone to resolve leukoplakia and erythroplakia in Fanconi anemia patients we had to discuss the need for a randomized controlled trial particularly under sample-size restrictions as very promising results were available from a single-arm clinical trial. Unfortunately, at a later stage, we had to suffer from the fact that single-arm clinical trials may sometimes mislead. When revisiting our planning at a later stage of a grant application, results of a randomized controlled trial had become available which were less impressive, but may still be of clinical interest. However, these results were perceived as disappointing in the light of previously raised hopes based on the results of the single-arm trial. We highlight some major problems when research is based on single-arm trials compared to randomized controlled trials. After debunking common arguments for the conduct of single-arm trials in rare disease we conclude that particularly in rare disease research should be based on randomized building blocks simply because more robust evidence is generated. The plea for single-arm trials should be substituted by a plea for cooperation of all stakeholders to provide best evidence for decision making under sample-size restrictions.

## Background

Randomized controlled trials (RCTs) with one control arm and at least one new treatment arm are the gold standard to inform about treatment efficacy, safety, and benefit-/risk-ratio. The re-emerging big data discussion reflects the hope that (unsystematic) large data collections can replace careful research into the efficacy and safety of treatments in certain (sub-)indications. In this context the use of historical data from registries or previous RCTS to replace a concurrent randomized control arm [1] is discussed. This approach essentially leads to the conduct of single-arm trials for which new methodology has been proposed to incorporate the historical information [2]. Researchers in the field of and patients suffering from rare diseases seem to be big advocates of such trials. This can be seen in a recent comparison of interventional clinical trials in rare and non-rare diseases, where it has been shown that the majority of trials in rare diseases are single-arm trials (63%) compared to 29% in non-rare diseases [3]. Strictly speaking it is only possible to evaluate the outcome of a single-arm trial, if the natural history (development/course) of disease is fully understood and constant over time so that it is fully clear that seemingly different outcomes beyond known prognostic factors between the experimental treatment and the historical control can only be caused by differences between treatments [4]. In other words, every time a single-arm trial is conducted, the researchers assume that the outcome in the control group (placebo or standard of care) is entirely known and not subjected to patient selection, or temporal effects. In consequence, relying on the counterfactual (the expected/hypothetical response in a control group) derived from registries or historical controls raises two issues:

1. The estimated difference between treatment and counterfactual might be biased and the potential drawbacks of using historical controls or registries have been discussed in many places [5, 6] and also apply here.
2. When the counterfactual is assumed to be known well enough and a single-arm trial is conducted, the underlying assumption of consistency of effects seen in the past and in the non-investigated control group is obviously not testable with the trial data. In case the control group is lacking, all hope is on circumstantial evidence to detect possible violations of the assumption.

It is worth noting that these issues are even more pronounced in rare diseases, where often less information from registries or prior studies is available. Even if information is available, it would obviously have lower precision than in frequent disease, and therefore, the counterfactual is squishier. As stated above, paradoxically, in rare diseases the proportion of single-arm trials is more than doubled compared to non-rare diseases [3].

In this paper, we are revisiting the planning of a randomized trial in a rare disease. In planning a clinical trial in Fanconi anemia (FA) we had to first discuss the need for randomisation and, unfortunately, at a later stage, suffer from the fact that single-arm clinical trials may sometimes mislead. We will first introduce the rare disease and the clinical evidence that was available to us in our planning meetings, before trying to explain all the hurdles that we faced while planning and a possible solution.

## A real-life example

FA is a rare inherited chromosomal instability syndrome characterized by congenital abnormalities, bone marrow failure and a predisposition to cancers including leukemia and solid tumors. Notably, the risk of head and neck squamous cell carcinoma (SCC) in FA is 500-fold higher in comparison with the general population [7]. FA-associated head and neck SCC is most commonly located in the oral cavity and surgery is the mainstay of therapy. Unfortunately, patients frequently present with late-stage disease and/or multiple lesions diminishing the utility of surgery. Still more, the role of adjuvant chemo- or radiotherapy for head and neck SCC in FA is limited because patients do not tolerate these therapies due to their underlying chromosomal instability. Not surprisingly, many FA patients relapse after therapy and eventually succumb to their cancer in less than 2 years after diagnosis [7]. Thus, systemic therapy at a premalignant stage of oral cancer appears to be a good option to prevent the development of an overt malignant tumor.

Patients with and without FA have premalignant oral lesions called leukoplakia, erythroplakia or leuko-erythroplakia and some of the lesions progress to oral SCC. Cellular and animal preclinical data suggested that pioglitazone, an oral hypoglycemic medication used in type 2 diabetes mellitus, may be beneficial for resolving these at-risk lesions. In an uncontrolled, open-label, single-arm clinical trial involving 21 non-FA patients administered 45mg of pioglitazone daily for 12 weeks, 15/21 (68%) of the patients showed a partial or complete involution of their oral premalignant lesions [8]. The estimated partial or complete spontaneous remission rate of oral leukoplakia, erythroplakia or leuko-erythroplakia in non-FA patients was less than 5% in the personal experience of our local oral surgeon. Given this information, the assumed treatment effect (the difference between observed treatment response and assumed counterfactual) was 63%.

With this prior knowledge, arguing for the conduct of an RCT in FA-patients is difficult. Arguments against randomization were the unwillingness of the patients to accept an inferior treatment when informed about the promising outcome of the single-arm clinical trial and the observed dramatic effect unlikely to be a pure chance finding. Despite little data on the natural course of disease, both, in FA- and non-FA patients, clinical equipoise was lost.

Subsequently, the results of a randomized placebo-controlled trial (*N* = 52) for the efficacy of 15 mg pioglitazone thrice daily for 24 weeks in non-FA patients became available. They showed a partial or complete response rate of 46% (12/26) in the pioglitazone group and 32% (8/25) response rate in the placebo group [9]. This RCT was terminated due to slow accrual after half of the planned sample size of 100 participants was recruited. Although in this randomized trial the treatment effect was estimated as 14%, compared with the impressive results from the single-arm clinical trial, the results were felt to be disappointing.

This perceived setback unfortunately prevented further discussion, e.g. whether a treatment effect of roughly 14% introduced by a rather non-toxic or debilitating treatment (as compared to surgery, or chemotherapy as the standard options for premalignant lesions or oral cancer) would indeed be an improvement large enough to continue research. In consequence, long-term effects not only on resolving leukoplakia and erythroplakia but also on oral cancer will not be evaluated.

## A case for RCTs in rare diseases

The striking differences in results between the single-arm and the randomized controlled trial in this instance underscore the notion that the assumption of knowing the counterfactual may be easily violated. By conducting a single-arm trial in an early development phase researchers implicitly or explicitly ignore this issue. Thereafter, planning an RCT based on the outcome in a single-arm trial most likely results in an overestimated treatment effect due to the missing adjustment for the outcome with the current standard or placebo, resulting in a trial underpowered to detect the true treatment effect. Even worse, the final decision could be based on perceived large treatment effects and further research to replicate the findings may not even be conducted.

Selection bias is one of the main sources responsible for overestimation (or seldom underestimation) of effect. If treatment is not randomly allocated between groups, there is no way of knowing which of the observed effect is due to treatment efficacy and which is due to population characteristics. A response rate of 68% for pioglitazone in the single-arm trial compared to a response rate for pioglitazone of 46% in the RCT might indicate that different populations have been studied. Most often patients with a more promising prognosis will be recruited for a single-arm trial. Whenever those prognostic factors that would describe the better patient prognosis are not recorded, not analyzed, or even not known, the single-arm trial is not able to identify these factors.

Controlled observational studies or, generally, study designs, which include some sort of control data without randomizing treatment allocation, most likely suffer from the same issue, because different populations might have been selected for the treatment and control group: patients with specific predispositions might (not) be allocated to the treatment group because a higher benefit (risk) is assumed [10] In contrast, randomization will “on average” balance all known and unknown confounders between treatment groups and the estimated treatment effect is adjusted for confounders by default.

A fundamental epistemological principle is proof of efficacy in a formal regimented setting; yet, current literature appears to rationalize the inability to complete RCTs. Particularly, in the field of rare diseases, there seems to be an increasing unwillingness to go for best evidentiary standards (e.g. [11–13]). The pioglitazone-example clearly demonstrates all the downsides of this approach.

It seems that not much has changed since 1975 in the discussion about clinical trial design [14] and unfortunately, the issue remains whether randomization is necessary, or can be avoided altogether (e.g. [15]). Often in rare diseases, there are arguments that limited sample size prohibits randomization or that outcomes will be dramatic and a single-arm trial will suffice to unequivocally substantiate the efficacy of a treatment. However, Prasad & Oseran concluded that for rare cancers, the issue of randomization (or lack of randomization) in clinical trials is more likely related to our expectations in the research community, rather than different tumor incidences [16]. If the above arguments hold true, there should be a proportionate correlation between disease incidence and the number of RCTs conducted in the disease. In rare cancers, the percentage of RCTs did not change even though there is a 6-fold difference in incidences of these cancers [17]. Similarly, the Institute for Quality and Efficiency in Health Care (IQWIG) concluded that approvals for orphan drugs, also in rare diseases, are largely based on conventional randomized designs so that the general feasibility is not in question [18]. Irrespective of reaching a pre-specified significance level, randomization leads to the best unbiased estimate of the treatment effect. Moreover, if the treatment effect is large, a small RCT will formally prove the effect.

## A decision making strategy based on RCTs

**Fig. 1:**
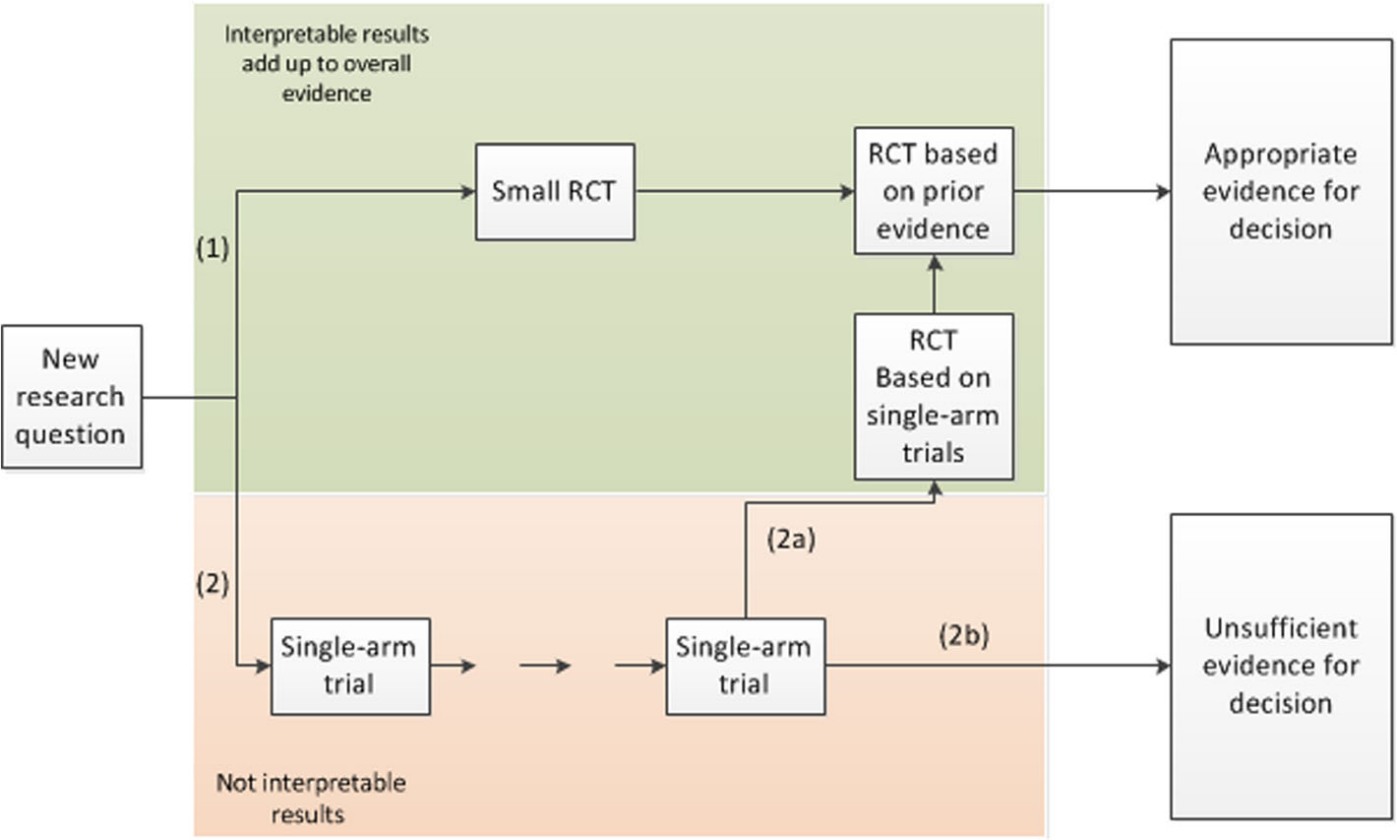
Global view of study planning

A comparison of decision-making strategies based on single-arm trials versus RCTs is outlined in Fig. 1. Particularly in those cases, where the natural history of the disease is not well understood, there is no good reason to attempt to answer a new research question utilizing a single-arm trial. Likewise, the risk that patient selection for a single-arm trial cannot be detected, speaks against this research approach. Often the discussion is centered on assessment of efficacy, but also the proper evaluation of safety is hampered by basing decision making on single-arm trials. Particularly in situations, where patients are multi-morbid and treated with many different drugs, it is difficult to attribute (and balance) effects without a control arm.

Strikingly and in contrast to the prevailing opinion, starting with an RCT from the beginning economizes patients (option 1). In addressing a new research question, a small RTC generates valuable data, which serves as a valid foundation for estimating the treatment effect unbiasedly and provides good information about the variability of this treatment effect as a basis for planning a next adequately powered RCT.

Particularly in the field of rare disease, the decision-making process may then be based on a meta-analysis of both RCTs (this approach would be in line with planning the trial as a two-stage adaptive design according to Bauer & Köhne [19]) including the first pilot and the definite trial. Even if the research question is not finally answered, the data from the two trials can complement each other and can eventually be combined with a future RCT. Here, the primary goal of the first RCT is not proof of efficacy by reaching a pre-specified significance level (for which the trial cannot be planned for properly with no prior information on the treatment effect variability available), but to establish the best possible basis for continuing research and, eventually, planning a second, properly powered RCT. Consequently, in reviewing the results of the first RCT most weight should be attached to the evaluation of the treatment effect estimate instead of focusing on the significance and dismissing further research, when non-significant results are seen. This idea is reflected in the two-stage adaptive design by Bauer & Köhne [19], where interim analysis results will be available that can be critically challenged, but most importantly be discussed with a limited competent audience to decide whether (a) the trial should proceed, (b) treatment modalities require some modifications to an extent that allows combining findings of the two stages or (c) to stop the trial because of “true” futility. Even in the last case, the trial is informative for future research in this area because it can provide a control effect (and its variability) for planning future trial that is relevant for a randomized situation and free from undetected selection bias towards patients with good prognosis.

When conducting a single-arm trial first (option 2), the resultant data is frequently less circumspect, treatment effects and effects of patient selection cannot be separated, which may lead to the conduct of another single-arm clinical trial and will eventually lead to decisions based on insufficient evidence (2b). In other instances data is regarded as inconclusive and an RCT is planned based on the single-arm clinical trials (2a). In addition to all previously mentioned problems of this approach, more assumptions are needed to include earlier stages of development with single-arm trials into the evaluation of this final RCT. In an extreme case, another RCT based on the first RCT may be necessary and interest may go lost.

Since route 2b does not cumulate appropriate evidence for decision-making, one can only consider routes 1 and 2a. While both strategies afford interpretable results, route 2a is less straight forward, a detour of sorts. Only route 1 accrues meaningful data throughout all clinical trials and the total sample size is less than compared with the sample size in route 2a. Only consequently starting research with RCTs ensures that all treated patients contribute to the overall evidence and no data is wasted. As our goal should be to base decision-making on unbiased estimates of the treatment effect, randomizing patients is the most economic approach. Following this strategy, RCTs can be interpreted as Lego®-type (randomized) building blocks that – similar to the children’s toy - can be combined and re-used creatively to craft stable (evidence) structures. The combination of effect estimates from RCTs in a meta-analysis, or the use as priors in a Bayesian analysis requires far fewer assumptions than the combination of results from single-arm trials. In a clinical decision making context combining RCT results requires the assumption of equal treatment effects (and not necessarily equal baseline event rates, or an identical structure of the patient population). In fact, this assumption is always made, whenever multi-center or multi-regional clinical trials are conducted and stratification for center or region is considered sufficient to account for potential structural differences between centres and regions in the concomitant setting. In contrast, combining and comparing of single-arm results is based on the assumption of equal effects, equal baseline rates, and equal structure in the combined/compared populations. All these are non-testable or assessable from the single arm trial.

By standardizing for baseline rates and the effect of background treatment in general, the Lego®-type (randomized) building blocks are combinable more flexibly. In instances, a fine-tuning may be needed in between the first and the second trial, but researchers are used to discuss, under which circumstances trials can be combined in a meta-analysis, or not. The randomized building may also be informative, if the intervention is modified to improve safety, but without any expectation that efficacy is influenced.

When illustrating the global view of study planning, we focus on single-arm trials as antagonists of RCTs. The underlying rationale, however, holds true for all study designs as antagonists, which include some sort of control data without randomizing treatment allocation, as well.

It is disputable, if and how well-planned observational studies and registries can play a part in our global view of study planning. We feel that observational research and particularly the implementation of disease registries, carefully planned and cautiously interpreted, is a must in situations, where information is sparse and therefore highly valuable. The availability of such data would improve the basis for planning of trials, help to assess general improvements of patient care and help to better guess, how many patients can likely be recruited for a certain clinical trial. In any case, avoidance of selection bias and representativity of the observed patients need to be addressed.

## Conclusions

While planning an RCT in FA, a rare disease, we encountered preferences from numerous stakeholders in favour of a single-arm trial. This preference was based on another single-arm trial where improvement of symptoms was seen that may have implications for the progression of disease. In the months of planning, it became clear that another randomized trial in the same condition and investigating the same drug was terminated due to a slow accrual. Although this trial has not been completed, it became clear, that an improvement of the symptoms was possible even under placebo and that the first single-arm trial had hugely overestimated the treatment effect. The unbiased estimate for the treatment effect would be clinically relevant if measured against the expected risks of the experimental treatment and the debilitating consequences of current treatment options for later stages of disease. In consequence, current knowledge should actually make the research approach attractive to confirm short-term- and long-term effects of treatment. Exaggerated hopes based on the single-arm trial, however, lead to disappointment and reservations against the idea to pursue the research approach. We assume that this is not the first example for research strategies that have been abandoned irrespective of whether there is a more promising or at least competing approach to be investigated, or not.

Due to these problems, we decided that a cautionary remark about the place of single-arm trials in today’s pharmacological decision-making process and research strategies was important. Moreover, when reading old discussions about this topic, we felt that particularly in rare disease there is merit in planning the research strategy based on RCTs as combinable building blocks that provide unbiased estimates of the treatment effect and, more importantly, avoid undocumented selection of patients.

Nothing has changed since Chalmers [14], Sibbald & Roland [5], and Haffner et al. [6]: Single-arm trials are linked to a wide field of problems, such as debatable interpretation, lower quality of evidence, unrecognized predictive or prognostic factors, and overestimation of the treatment effect derived from a comparison to effects seen outside the current trial. The conduct of RCTs suffers from reluctance on both, the researchers’ and the patients’ side in planning and participating in an RCT if an apparent effect has been observed in the single arm trial. Often, however unlikely in rare disease, an RCT is only conducted after several single-arm trials, if these lead to obvious heterogeneity and the need of an RCT becomes inevitable. While on a micro-level it might seem that single-arm trials are the better choice in diseases with limited patient recruitment, this renders the accrual-argument of single-arm proponents invalid and speaks for planning research strategies with randomized building blocks.

Even though it is clear that research needs to start somewhere and it is probably not possible to literally follow Chalmers’ advice, particularly in rare disease, the risk that planning a single-arm trial may generate wasteful information that is, at best, difficult to be used in future research should be seen as a disincentive for single-arm trials. Considering in addition, that most of the efforts to conduct drug trials lie in the formal need to agree to the protocol with various stakeholders clarifies that the additional investment of implementing a randomisation and introducing a control is probably the smallest part in the discussion.

## Abbreviations

FA: Fanconi anemia
IQWIG: Institute for quality and efficiency in health care
RCT: Randomized controlled trial
SCC: Squamous cell carcinoma
